# Chromophobe renal cell carcinoma associated with eosinophilia: A report of three cases

**DOI:** 10.3892/etm.2014.1725

**Published:** 2014-05-20

**Authors:** YONG-BAO WEI, BIN YAN, ZHUO YIN, JIN-RUI YANG

**Affiliations:** Department of Urology, The Second Xiangya Hospital, Central South University, Changsha, Hunan 410011, P.R. China

**Keywords:** chromophobe renal cell carcinoma, eosinophilia, recurrence

## Abstract

Eosinophilia is typically associated with allergic reactions, parasitic infestations, certain forms of vasculitis, the use of certain medications and hematologic malignancies. In addition to eosinophilia associated with gastrointestinal tumors, lung cancer and thyroid carcinoma in solid malignancies, there are a limited number of cases describing peripheral hypereosinophilia in urologic tumors. The present study reports three cases of eosinophilia in patients with chromophobe renal cell carcinoma (CRCC) and investigates the association between excessive eosinophilia and the recurrence and prognosis of renal carcinoma. This is the first report of CRCC associated with excessive eosinophilia. Eosinophilia following tumor resectioning may indicate a poor prognosis, tumor recurrence and rapid disease progression.

## Introduction

Eosinophilia is typically associated with allergic reactions, parasitic infestations, certain forms of vasculitis, the use of medication and hematologic malignancies. However, eosinophilia in solid malignancies is rarely reported. Severe peripheral hypereosinophilia has been reported in solid tumors including gastrointestinal tumors (1), lung cancer (2,3), and thyroid carcinoma (4). However, there have been a limited number of reports describing peripheral hypereosinophilia in urologic tumors (5,6). Todenhöfer *et al* (6) presented the first and only case of severe paraneoplastic hypereosinophilia in a patient with renal cell carcinoma. The present study reports three cases of eosinophilia in patients with chromophobe renal cell carcinoma (CRCC) and investigates the association between excessive eosinophilia and the recurrence and prognosis of renal carcinoma.

## Case reports

### Patient history

Between September 2010 and September 2013, three patients (two males and one female) were admitted to The Second Xiangya Hospital (Changsha, China). Two patients, one male and one female, presented with lumbago. Another male patient complained of weight loss of 5 kg in three months. All the patients were found to have a kidney mass according to a computed tomography (CT) scan of the abdomen and were diagnosed with a renal tumor (Table I). One patient complained of a weight loss of 5 kg in three months. None of the patients exhibited hematuria or a fever. Patient medical histories were negative for specific drug use, food allergies, parasitic infestations and exposure to tuberculosis. Informed consent was obtained from the patient’s families.

### Patient examination

**Figure 1:**
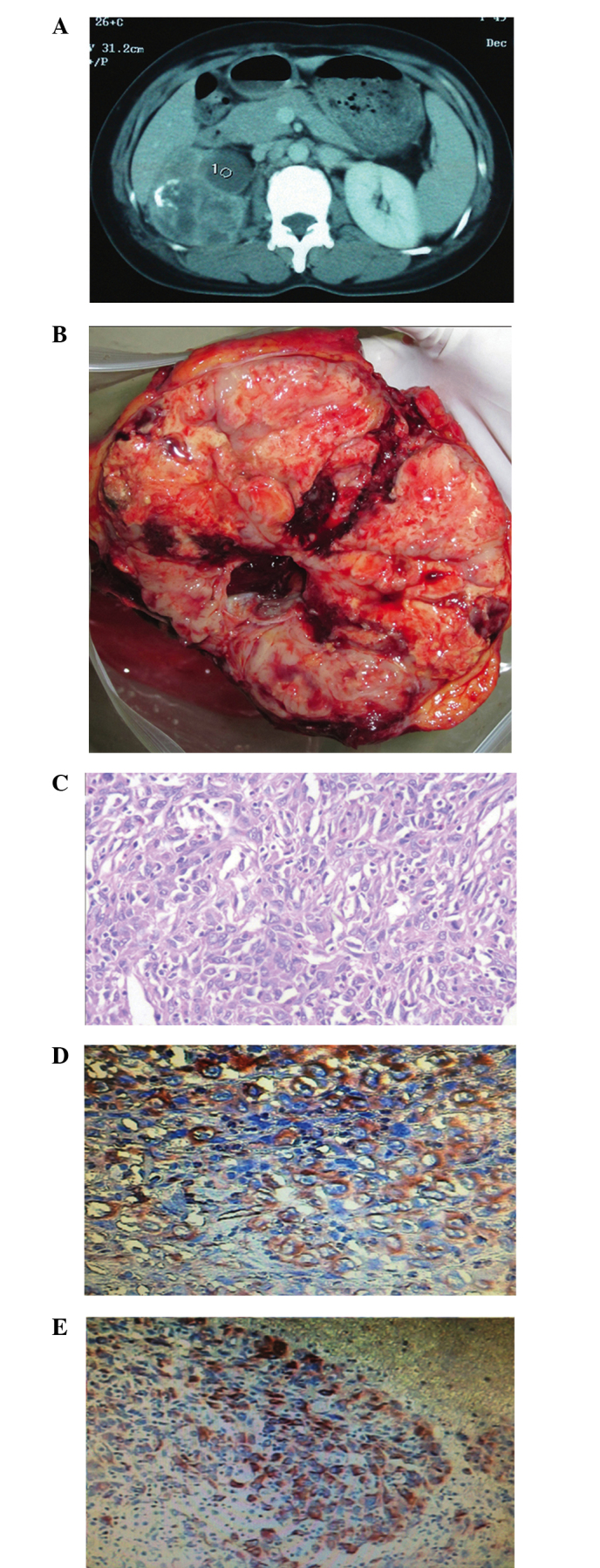
Female patient 3. (A) Computed tomography scan demonstrating a 7.5 cm solid cystic mass in the right kidney with necrosis and calculus. (B) Cross section of the resected kidney revealing the tumor tissue. (C) H&E staining; magnification, ×100. (D and E) Immunohistochemical staining: vimentin (VIM^+^) and cytokeratin (CK^+^), respectively, of renal tumor tissue showing chromophobe renal cell carcinoma of eosinophilic variant with sarcomatoid components (magnification, ×100). H&E, hematoxylin and eosin.

Physical examination revealed that each patient had a soft and flat abdomen. No masses were located in the abdominal region and the superficial lymph nodes were not palpable. Stools were negative for ova and parasites. Rheumatoid immune factors including C-reactive protein, procalcitonnin, antinuclear antibody and anti-neutrophil cytoplasmic antibody were all negative in the blood. Tuberculosis tests were negative. Tumor markers and alkaline phosphatase were negative in the blood. A bone marrow biopsy was performed and demonstrated no evidence of leukemia. The chest X-ray demonstrated no positive signs of pulmonary and metastatic lesions. A CT scan of the abdomen and pelvis of the female patient revealed a kidney tumor without venous tumor thrombus and distant metastasis (Fig. 1). One retroperitoneoscopic radical nephrectomy and two open surgeries were performed without any complications. Swollen lymph nodes were not observed between surgeries. Histological and pathological examinations revealed CRCC. Eosinophilic variant CRCC with sarcomatoid components was observed in the female patient (Fig. 1).

### Outcomes

**Figure 2:**
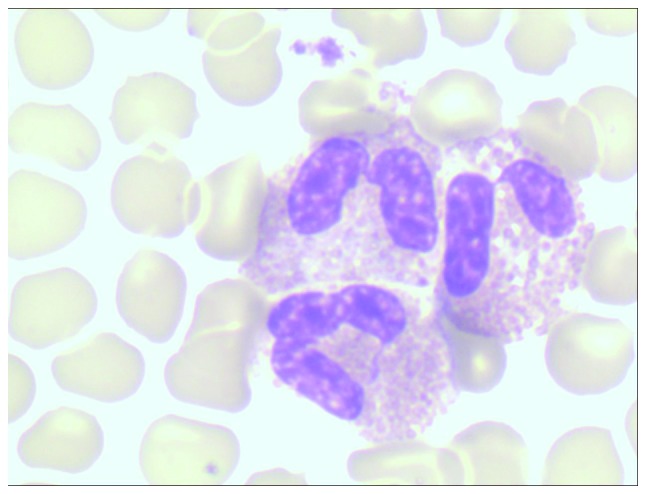
Peripheral blood smear demonstrating eosinophilia.

**Figure 3:**
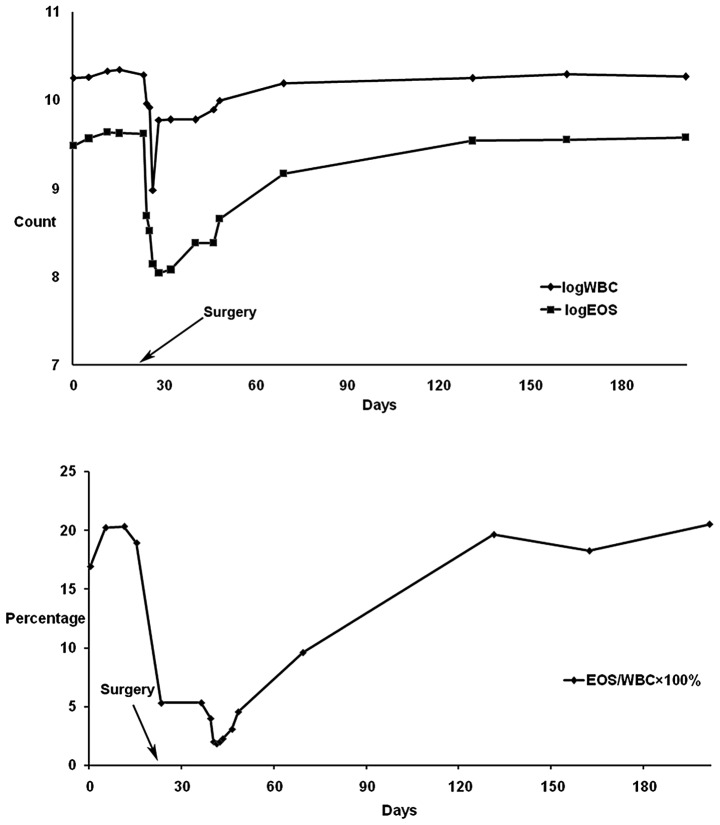
Graphs showing (A) changes in the count of eosinophilic granulocytes and leukocytes (logs) with time, and (B) the percentage of eosinophilic granulocytes for patient 3.

Prior to surgery, routine blood tests revealed that all patients had persistent leukocytosis (11,360–22,230/μl, normal range: 4,000–100,000/μl) and eosinophilia (1,120–5,060/μl, normal range: 0–800/μl; Fig. 2). A routine blood test was administered during the perioperative period and at the one-year follow-up following discharge. Eosinophilia disappeared in the first month following radical nephrectomy. The two male patients presented with normal eosinophilic granulocytes without tumor recurrence following surgery and at the one-year follow-up. However, blood analysis of the female patient revealed that the leukocyte and eosinophilic granulocyte counts had gradually increased and returned to the preoperative levels (Fig. 3). Ultrasound examination revealed renal carcinoma recurrence in the original renal region involving the abdominal wall and intestine. Blood tests demonstrated that the levels of eosinophilic granulocytes continuously increased (19.61–20.49%, normal range: 0–5%). The patient suffered from pain, fever and an abdominal mass. The female patient succumbed to the disease six months following surgery due to tumor recurrence.

## Discussion

Marked peripheral eosinophilia associated with neoplasia is rare and is observed in ~0.5% of all documented malignancies, most frequently in hematological malignancies (1). Peripheral eosinophilia associated with neoplasia is more common in patients with renal carcinoma. To the best of our knowledge, this is the first report of CRCC associated with excessive eosinophilia. Following the exclusion of other causes, such as infections, allergies, collagen disease, vascular diseases and concomitant malignant hematopoietic diseases, it was considered to be a paraneoplastic manifestation. The majority of reports have indicated that this phenomenon is associated with a poor prognosis and rapid disease progression (2,6).

Eosinophilia may disappear following tumor removal and reappear with tumor relapse or dissemination. In the three patients in the present report, leukocytosis and eosinophilia normalized following radical nephrectomy. However, the levels of eosinophilic granulocytes relapsed to preoperative levels and tumor recurrence was observed in the female patient one month following surgery. Since no other causes of peripheral eosinophilia were identified, the authors conclude that renal carcinoma-associated blood eosinophilia indicated a recurrence and poor prognosis of CRCC.

CRCC is a distinctive subtype of renal cell carcinoma and is classified into two variants, typical and eosinophilic, where the prognosis is more favorable for patients with the eosinophilic rather than the typical variant (7). Clinical evidence indicates that CRCC is one of the less aggressive types of renal cell carcinoma, but it has been accepted that sarcomatoid changes are an unfavorable sign histologically (8). Several previous studies have reported the phenomenon of tumor-associated tissue eosinophilia (TATE) at the tumor site (9,10). TATE may occur together with or separately from tumor-associated blood eosinophilia (TABE). The association between TATE and TABE remains unclear. Notably, tumors with TATE alone appear to have a better prognosis compared with those without TATE, while TABE is associated with tumor spread and a poor prognosis (11). The causes of this difference remain unclear and require identification. In the present study, TABE was present but TATE was not observed at the tumor site of the patients. Consistent with numerous reports, the female patient with sarcomatoid changes and TABE exhibited a poor prognosis and succumbed to tumor recurrence (2,6,13).

The pathogenesis of TABE remains unclear. Numerous mechanisms have been postulated, including bone marrow stimulation via circulatory factors produced by the tumor itself, tumor necrosis as the promoter of increasing eosinophils and stimulation of eosinophil production resulting from the seeding of metastatic neoplastic cells to bone marrow (12). The theory of bone marrow stimulation via circulatory factors has been acknowledged (4,14). Cytokines including interleukin-3, interleukin-5 and granulocyte-macrophage colony-stimulating factor produced by the primary tumor have been considered to account for increased eosinophilic granulocytes (1,3,4,13). Although these cytokines were not measured in the three patients, eosinophilia was considered to be associated with the increased cytokine levels. However, there is no single mechanism for this phenomenon as the correlation between disseminated carcinomas, hypereosinophilia and cytokine production is complex and unknown.

The role of eosinophils in tumors requires further investigation. The presence of eosinophils has been associated with favorable prognosis in certain studies but poor prognosis in other studies (9). The authors speculate that the role of eosinophils associated with solid tumors may alter with time and location. Initially, TATE is a particularly specific reaction in certain tumors and the reactive eosinophils provide protection against tumor cells. As the infiltration and invasiveness of the tumor cells increase, eosinophils lose their protective role and move to the peripheral blood. As a result, tissue eosinophilia shifts to blood eosinophilia, indicating an advanced stage of the neoplasm. The difference between tissue eosinophilia and blood eosinophilia, in addition to the hypothetical shifting process, remains unidentified. The underlying mechanism requires validation through additional studies.

In conclusion, this is the first report of CRCC associated with excessive eosinophilia. Eosinophilia following tumor resection may indicate a poor patient prognosis, tumor recurrence and rapid disease progression. The role of the eosinophil in renal tumors and the mechanism of tumor-associated blood eosinophilia require further elucidation.

## Figures and Tables

**Table I:** General information of the three patients diagnosed with chromophobe renal cell carcinoma with eosinophilia.

			Tumor					Mean percentage of eosinophils		
				
Patient	Gender	Age (years)	Position	Size (cm)	TNM	Recurrence	Metastasis	Pre-surgery	Month 1 after surgery	Months 2–12 after surgery
1	Male	53	Right kidney	7.0	T1bN0M0	No	No	17.53	2.86	2.54
2	Male	56	Left kidney	6.2	T1bN0M0	No	No	12.35	2.77	2.63
3	Female	48	Right kidney	7.5	T2aN0M0	Yes	Yes	19.08	3.37	16.98

Mean percentage of eosinophils = eosinophil count/leukocyte count × 100; TNM, tumor node metastasis, the TNM stage was based on the 7th American Joint Committee on Cancer (AJCC 2010). Size is the maximum diameter of the tumor.
